# Intragenic transcriptional *cis*-activation of the human immunodeficiency virus 1 does not result in allele-specific inhibition of the endogenous gene

**DOI:** 10.1186/1742-4690-5-98

**Published:** 2008-11-04

**Authors:** Alex De Marco, Chiara Biancotto, Anna Knezevich, Paolo Maiuri, Chiara Vardabasso, Alessandro Marcello

**Affiliations:** 1 Laboratory of Molecular Medicine, International Centre for Genetic Engineering and Biotechnology (ICGEB), Padriciano, 99, 34012 Trieste, Italy; 2 IFOM-IEO, Milan, Italy; 3 EMBL, Heidelberg, Deutschland, Germany; 4 Laboratory of Molecular Virology, International Centre for Genetic Engineering and Biotechnology (ICGEB), Padriciano, 99, 34012 Trieste, Italy

## Abstract

**Background:**

The human immunodeficiency virus type 1 (HIV-1) favors integration in active genes of host chromatin. It is believed that transcriptional interference of the viral promoter over the endogenous gene or vice versa might occur with implications in HIV-1 post-integrative transcriptional latency.

**Results:**

In this work a cell line has been transduced with a HIV-based vector and selected for Tat-inducible expression. These cells were found to carry a single silent integration in sense orientation within the second intron of the *HMBOX1 *gene. The HIV-1 Tat transactivator induced the viral LTR and repressed *HMBOX1 *expression independently of vector integration. Instead, single-cell quantitative *in situ *hybridization revealed that allele-specific transcription of *HMBOX1 *carrying the integrated provirus was not affected by the transactivation of the viral LTR in *cis*.

**Conclusion:**

A major observation of the work is that the HIV-1 genome has inserted in genes that are also repressed by Tat and this could be an advantage for the virus during transcriptional reactivation. In addition, it has also been observed that transcription of the provirus and of the endogenous gene in which it is integrated may coexist at the same time in the same genomic location.

## Background

Retroviruses, such as human immunodeficiency virus type 1 (HIV-1) require reverse transcription and integration into host chromatin to establish a provirus as an obligatory replication step. The choice of the integration site is a crucial intermediate of the virus life cycle. The chromatin context determines the efficiency of viral transcription and is involved in the establishment of post-integrative latency that is the major obstacle to HIV-1 eradication with current antiviral therapies [1-3]. In addition, insertion of a provirus in the human genome can cause several adverse effects [4]. For example, insertion of the retrovirus close to a proto-oncogene may induce transformation of the cell. Gene therapy approaches suffer most from these effects and recently it has been demonstrated that the activation of an oncogene caused transformation in several children treated with a therapeutic retroviral vector [5]. In principle, insertion of an ectopic transcription unit within a gene may also result either in disruption of exonic sequences, introduction of alternative splicing or transcriptional interference. Clearly, these negative effects would increase in importance relative to the increasing unbalance of the endogenous gene expression between alleles.

Integration site selection by retroviruses is not sequence-specific but also not random. HIV-1 favors integration within active transcription units [6-8]. Additional features are the requirement of host factors such as the lens epithelium-derived growth factor LEDGF/p75 for efficient targeting of active transcription units [9] and a DNA substrate wrapped around nucleosomes. Indeed, integration of HIV-1 is linked to nucleosomal markers of active transcription (H3/H4 acetylation, H3K4 methylation) and negatively correlated with inhibitory modifications (H3K27 trimethylation and DNA CpG methylation) [10]. Subtle differences in the integration site choice exist among retroviruses. Murine leukemia virus (MLV) integrates within highly active promoters at ± 5 kb from the transcription start sites [7,11]. HIV-1 instead, although also favoring active genes, does not show a preference for promoter-proximal integration. Rather, the virus inserts throughout the transcriptional unit with a bias towards intronic sequences: this is the likely result of the greater size of introns compared to exons within a gene [6].

A crucial aspect of HIV-1 pathogenesis is the control of provirus transcription. In particular the ability of the virus to maintain a reservoir of transcriptionally silent proviruses in resting memory T cells for long periods of time. Multiple mechanisms have been postulated to concur in these processes. Host factors, for example, may be limiting the activity of the Tat transactivator. Tat interacts with a *cis*-acting RNA element (trans-activation-responsive region; TAR) present at the 5' end of each viral transcript [12]. Through this interaction, the protein activates HIV-1 transcription by promoting the assembly of transcriptionally active complexes at the LTR through multiple protein-RNA and protein-protein interactions [13]. Tat interacts with the core RNA polymerase II [14,15], the TATA-binding protein associated factor (TAFII) [16], TFIIH [17], cyclin-dependent protein kinase 7 [18], SP1 [19], nuclear factor of activated T cells (NFAT) [20], several histone acetyltransferases [21-23] and cyclin T1 [24]. On the other hand, the chromatin context at the site of integration should determine whether the provirus is transcriptionally active, poised for activation or inactive [25]. Early studies showed that latency involved integration into regions of heterochromatin [26,27]. More recent systematic genome-wide analysis of the chromosomal features negatively associated to HIV-1 transcription revealed that low levels of LTR-driven expression correlated with integration in gene deserts and in centromeric heterochromatin, but also in highly expressed cellular genes [28]. Furthermore, HIV-1 has been found in intronic regions of actively transcribed genes in resting memory CD4+ cells derived from patient on highly active antiretroviral treatment [29]. The paradox of HIV-1 integration in active genes while being transcriptionally silent requires molecular investigation of the phenomenon. Unfortunately most cellular models of HIV-1 post-integrative latency harbor the provirus outside of transcribed genes [3]. In this work a cell-line that carries a single repressed provirus integrated within the active transcription unit of the *HMBOX1 *gene has been generated. Tat-mediated induction of provirus transcription resulted in the inhibition of *HMBOX1 *expression. However, this effect could be ascribed to Tat expression and not to activation of the viral LTR. Indeed, a subset of activated cells showed bi-allelic expression of *HMBOX1 *together with expression of the provirus within one of the alleles. These results are discussed in light both of HIV-1 pathogenesis and of the potential use of lentiviral vectors for gene therapy applications.

## Results

### Generation and characterization of a cell line carrying a stably integrated lentiviral vector

**Figure 1:**
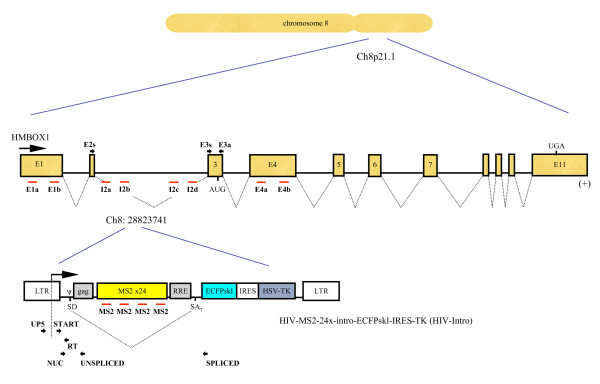
**Genomic organization of the HMBOX1 gene and of the HIV-intro construct in HOS_A4 cells**. Position of the RT-PCR primers are indicated by black arrows. Positions of the FISH probes are indicated by red bars.

The HIV-Intro-MS2 × 24-ECFPskl-IRES-TK lentiviral vector (for simplicity: HIV-Intro) has been engineered to contain the elements required for RNA production: the 5' LTR, the major splice donor (SD1), the packaging signal Ψ, the RRE, the splice acceptor A7 and the 3' LTR that drives 3'-end formation (Figure 1). The construct carries also an array of 24 repeats of the MS2 phage coat protein within the intron, to increase specific detection of nascent mRNA, a reporter of gene expression (ECFP) fused to the peroxisome localization signal Ser-Lys-Leu (skl) and the selectable marker thymidine kinase (TK) of herpes simplex type 1.

**Figure 2:**
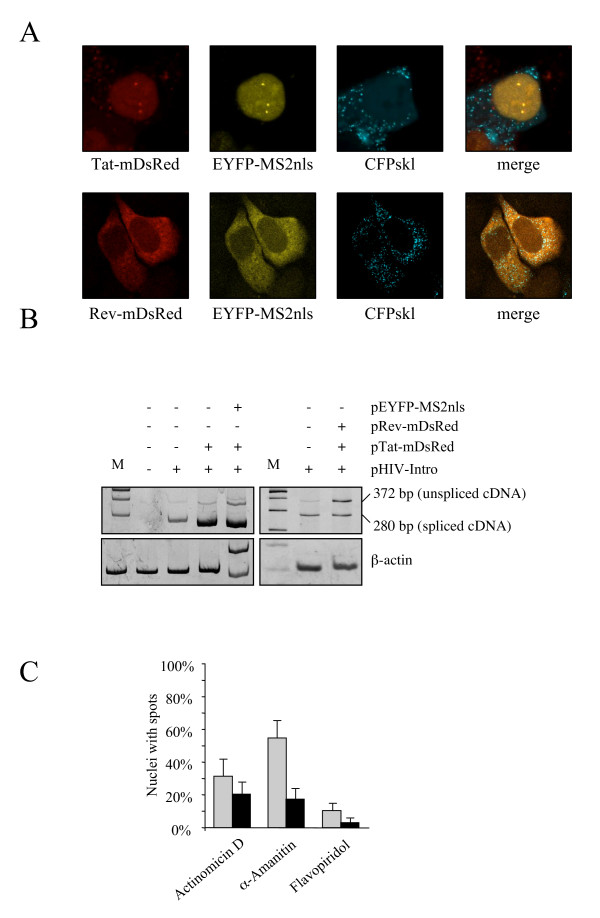
**A) HeLa cells were cotransfected with pHIV-Intro, pEYFP-MS2nls and either mDsRed tagged Tat (top) or Tat and mDsRed tagged Rev (bottom). **Yellow spots in the nucleus correspond to nascent RNA from transfected plasmids. Cyan spots in the cytoplasm correspond to ECFPskl localized to peroxisomes.** B)** RT-PCR on HeLa cells transfected as indicated. Three primers were used, their position is shown in Figure 1. Resulting bands correspond to the unspliced and spliced HIV-Intro RNAs. Bottom panels: β-actin loading control (M = molecular weight marker). **C)** Effect of RNAPII inhibitors on HIV-Intro transcription in transfected HeLa cells transfected as indicated in Figure 2A, top panels. Nuclei showing transcription spots were scored 1 hour (gray bars) an 6 hours (black bars) after treatment with Actinomicin D (10 μg/ml), α-Amanitin (10 μg/ml) or Flavopiridol (500 μM).

In order to characterize this construct extensively before transduction, HeLa cells were transfected with plasmid HIV-Intro together with a plasmid expressing a monomeric DsRed-tagged Tat Figure 2A, top panels). As expected from previous studies showing transcribed nascent RNA by MS2-tagging [30,31], bright yellow spots appeared within the nucleus. Each spot corresponds to several plasmids clustered together that express viral RNA [32]. As expected, Tat was found at transcription sites because it binds the 5'-end of each transcript. The reporter of gene expression ECFPskl was found in the cytoplasm. When a plasmid expressing a DsRed-tagged Rev was co-transfected together with Tat (without tag), the unspliced RNA was found in the cytoplasm, consistent with its Rev-mediated export (Figure 2A, bottom panels). These results are mirrored by the behavior in RT-PCR using a set of primers that distinguish pre-mRNA from spliced RNA. As shown in figure 2B, basal transcription is up-regulated by Tat with a higher proportion of spliced over unspliced RNA. Co-transfection of a plasmid encoding pEYFP-MS2nls does not affect the splicing reaction, ruling out perturbation of the system by such a strong RNA binding protein. Expression of Rev instead increased the proportion of unspliced RNA, consistent with its role in RRE-containing RNA stabilization and export.

A key question that arose while doing these experiments was the real nature of these yellow spots in the nucleus (Figure 2A, top panels). To confirm that these where sites of HIV-Intro transcription we incubated the cells with inhibitors such as Actinomicin D, α-Amanitin or Flavopiridol. As shown in Figure 2C, a rapid decrease of the number of transcription spots was observed with all three inhibitors. Hence, RNA-dependent accumulation of RNA at these sites was dependent on RNAPII activity.

**Figure 3:**
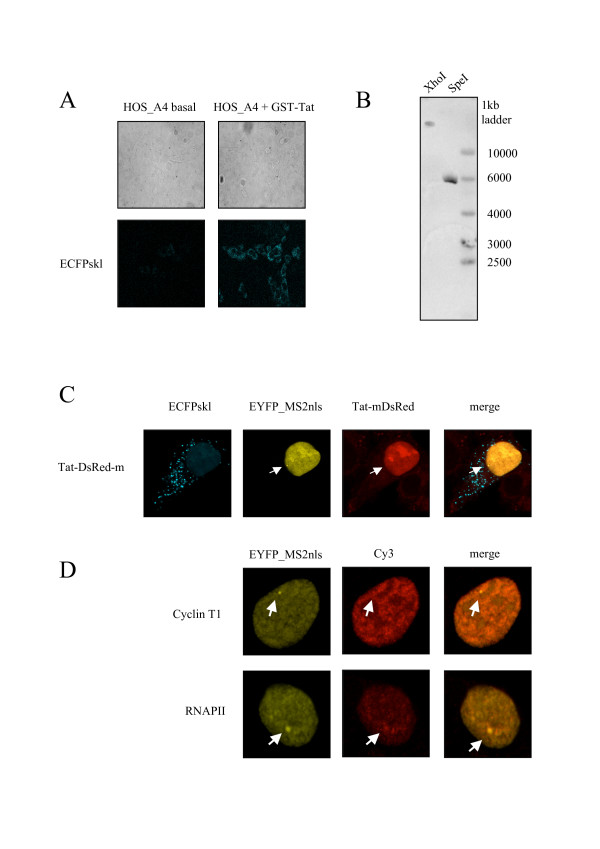
**A) Generation of HOS_A4 cells by transduction with HIV-Intro and selection as described in the text.** Tat induction induced the expression of ECFPskl in the cytoplasm. Top panels: phase contrast. Bottom panels: ECFP channel. **B)** Southern blot analysis of HOS_A4 cells shows the presence of a single integration event. Genomic DNA was digested with XhoI or SpeI and hybridized with a probe encompassing ECFP. **C)** Effect of Tat-mDsRed on HOS_A4 cells. Co-localization of Tat and HIV-Intro RNA is shown on the single transcription spot present in HOS_A4 cells. Correct gene expression is demonstrated by the ECPFskl signal in the cytoplasm. **D)** Co-localization of RNAPII and Cyclin T1 on HOS_A4 transcription spots. Cells were transfected with pEYFP-MS2nls and Tat, fixed and Cyclin T1 (top panels) or RNAPII (bottom panels) detected by immunofluorescence as described in [30].

Next a strategy was designed to express the HIV-Intro construct from a single chromatinized location in a Tat-inducible way. Osteosarcoma HOS_143b cells, that are negative for thymidine kinase activity (TK-), were transduced with the HIV-Intro vector pseudotyped with the VSV-G envelope. To select for clones that carry an inducible integrated provirus, cells that constitutively expressed high levels of HSV-TK were selected against by treatment with 50 μg/ml ganciclovir. Surviving cells, that were either non-transduced, or transduced but with a low level of TK expression, were treated with GST-Tat and briefly selected for inducible HSV-TK expression in hypoxanthine, aminopterin and thymidine (HAT) medium. Clonal populations were obtained by limiting dilutions and colonies were visually scored for low basal level of ECFP expression in the cytoplasm and to be highly inducible by GST-Tat by fluorescence microscopy. The HOS_A4 cell clone showed a robust and homogenous induction of ECFPskl in the cytoplasm upon treatment with GST-Tat (Figure 3A). These cells were transfected with plasmids encoding EYFP-MS2nls and Tat-mDsRed. As shown in figure 3C, HOS_A4 showed ECFPskl in the cytoplasm and presented one single bright yellow spot in the nucleus compatible with a single site of HIV-Intro transcription that co-localized with Tat-mDsRed. Immuonfluorescence with antisera against Cyclin T1, the P-TEFb component recruited directly by Tat on the viral RNA, or RNAPII demonstrated enrichment of such factors at these sites (Figure 3D).

These results are compatible with one integration event of the HIV vector in HOS_A4 cells. Indeed, analysis by southern blotting (Figure 3B) and cloning of the integration sites by inverse PCR revealed that the provirus lay within the *HMBOX1 *(homeobox containing 1) cellular gene.

### Allele-specific expression of *HMBOX1 *following HIV-Intro-MS2 × 24-ECFPskl-IRES-TK transactivation

**Figure 4:**
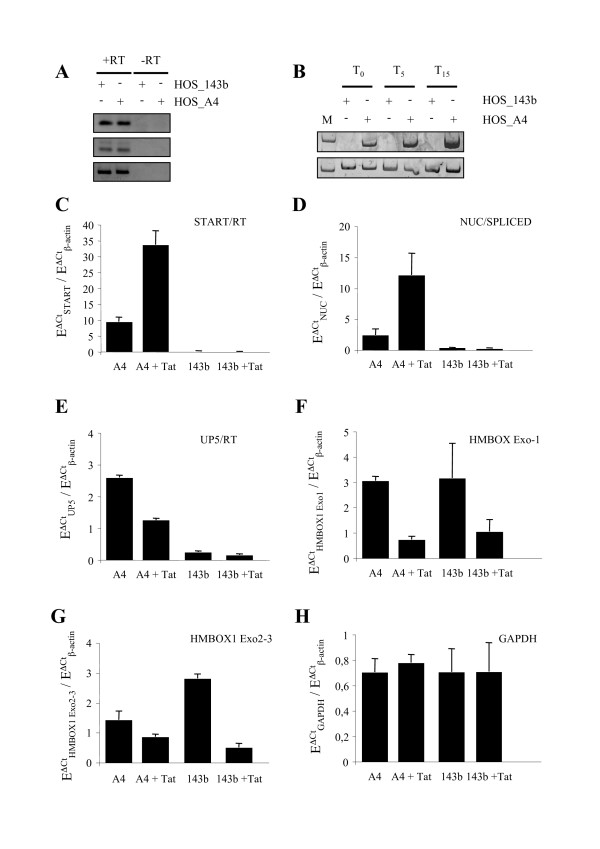
**A) RT-PCR analysis of *HMBOX1 *expression in parental HOS_143b and clone HOS_A4 using primers for *HMBOX1 *Exon 3 (79 bp, top panel) and Exon 2/Exon 3 splicing (86 bp, middle panel).** Bottom panel: β-actin control (230 bp). **B)** RT-PCR analysis of HIV-Intro expression (280 bp top panel) in parental HOS_143b and clone HOS_A4 using primers HIV_SPLICED and HIV_NUC (Table 1). Bottom panel: β-actin control (M = molecular weight marker; T = hours after Tat induction). C-H) Quantitative RT PCR for HIV-Intro, *HMBOX1 *and *GAPDH *expression using the indicated primers shown in Figure 1 and Table 1. Each histogram is the mean of three experiments normalized for β-actin expression and corrected for primer efficiency (E = 10^-1/slope^).

Human *HMBOX1 *is composed of 11 exons, spanning about 160 kb within chromosome 8 p21.1 (Figure 1). *HMBOX1 *is believed to encode for a transcription factor involved in the transcriptional regulation of key eukaryotic developmental processes. *HMBOX1 *is widely expressed in pancreas and the expression of this gene can also be detected in pallium, hippocampus and hypothalamus [33]. In HOS_A4 cells the HIV-Intro lentiviral vector integrated within the second giant intron of *HMBOX1 *(Figure 1). In order to assess expression of *HMBOX1 *in HOS cells RT-PCR was performed with primers specific for Exon3 or encompassing Exon2/Exon3 junction (Figure 4A). Both parental HOS 143b and HOS_A4 expressed *HMBOX1 *at similar levels. However, this assay was neither quantitative nor specific for the *HMBOX1 *allele carrying the integrated vector. A similar approach was also employed for the HIV-Intro transcript. As shown in Figure 4B, a basal level of HIV-Intro expression was detected in HOS_A4 that could be up regulated by Tat transfection.

In order to detect allele-specific transcription in the *HMBOX1 *locus carrying the integrated provirus a quantitative RT-PCR was developed according to the protocol of Han and collaborators [29]. As shown in the diagram of Figure 1, RT-PCR primers were designed to detect also *HMBOX1 *transcripts containing HIV sequences upstream of the viral transcription start site. RNA from HOS_A4 cells was reverse-transcribed and the resulting cDNA was amplified with two primers that share the HIV_RT primer. HIV_UP5 amplifies only HIV-Intro sequences produced as a result of transcription of *HMBOX1 *reading through the HIV-Intro genome that is inserted into the gene. Because the forward primer is located upstream of the transcription start site and the reverse primer is located downstream of the LTR, only RNA species initiating upstream of the HIV-1 transcription start site could be amplified. HIV_START instead is able to amplify any HIV-Intro transcript that has initiated at the viral start site. To prevent amplification from HIV-1 DNA, isolated RNA was treated with DNase before RT-PCR. In addition, control reactions from which RT was omitted were included in each experiment and were invariably negative. A positive control of HIV transactivation involved a set of primers for the HIV-1 spliced RNA product (primers HIV_nuc and HIV_spliced). PCR amplification was conducted in the presence of the dye CyberGreen for relative quantification of PCR products. Transfection of Tat induced HIV-1 transcripts several folds with both primer sets detecting HIV-1 transcripts (Figure 4C and Figure 4D). This result is perfectly in line with the well-known Tat-transactivation of the viral LTR and with RT PCR data shown in Figure 4B[34]. Allele-specific detection of *HMBOX1 *RNA instead showed a marked decrease in response to Tat (Figure 4E). This result would be explained by negative interference with the expression of *HMBOX1 *due to activation of a strong promoter embedded within the gene. However, when the analysis was conducted on two primer sets specific for the *HMBOX1 *gene both in HOS_A4 and the parental HOS_143b we realized that expression of *HMBOX1 *was affected by the presence of Tat *per se *and not by the activation of the viral LTR (Figure 4F and Figure 4G). This effect was not a general effect on transcription since the *GAPDH *gene was not affected (Figure 4H).

### Single-cell analysis of *HMBOX1 *and HIV-1 expression

Ensemble-averaged analysis such as RT-PCR that relies on the evaluation of a number of cells does not allow distinction between expression of each *HMBOX1 *allele. In fact, although we analyzed allele-specific expression of the allele carrying the provirus, still we don't know whether *HMBOX1 *expression was balanced between alleles or not.

In order to evaluate the simultaneous expression of both *HMBOX1 *alleles and of the integrated provirus, transcripts were detected by quantitative fluorescent *in situ *RNA hybridization (FISH). The amount of RNA on the transcription spot is determined by the rate of transcription and the rate of RNA processing. At steady state it could be derived from the intensity of the fluorescence signal compared with the intensity of the signal from a known reference as described previously [30]. For this purpose for each probe and each acquisition we prepared a calibration curve spotting different amounts of probe on a coverslip in a constant volume. The probes were acquired and deconvoluted using the same conditions used for the samples (see Methods). The z-projection sum of all planes was averaged and this value represent the signal emitted by each amount of probe. Therefore, the number of probes for each voxel (a volume pixel in a three-dimensional image) could be calculated for each point of the calibration curve. In the case of HIV-Intro transcripts, the number of RNAs on the transcription spot in the presence of Tat was calculated to be 17 ± 4.

**Figure 5:**
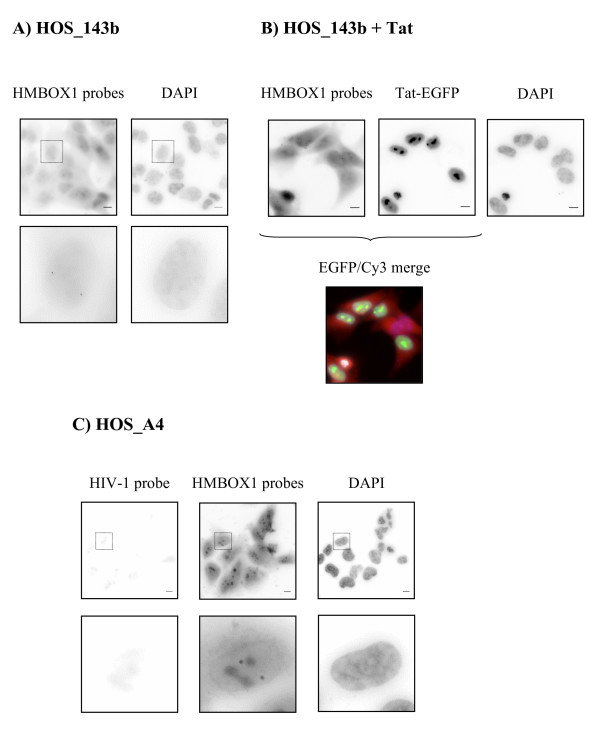
**A) FISH for *HMBOX1 *RNA on parental HOS_143b cells.** Top panel: large field image (bar = 10 μm). Bottom panels: single cell from the figure above (inset). Two distinct hybridization signals per nucleus demonstrate bi-allelic expression of the *HMBOX1 *gene. **B)** Same as A after transfection of Tat-EGFP. Also the signal for Tat-EGFP is shown (middle panels). Bottom panel: merge of *HMBOX1 *hybridization and Tat-EGFP expression. **C)** FISH for HIV-Intro RNA on HOS_A4 cells. Top panel: large field image (bar = 10 μm). Bottom panels: single cell from the figure above (inset). Absence of the hybridization signal with the HIV probe is due to silencing of the gene without Tat (left panels). Two distinct hybridization signals per nucleus demonstrate bi-allelic expression of the HMBOX1 gene (middle panels).

**Figure 6:**
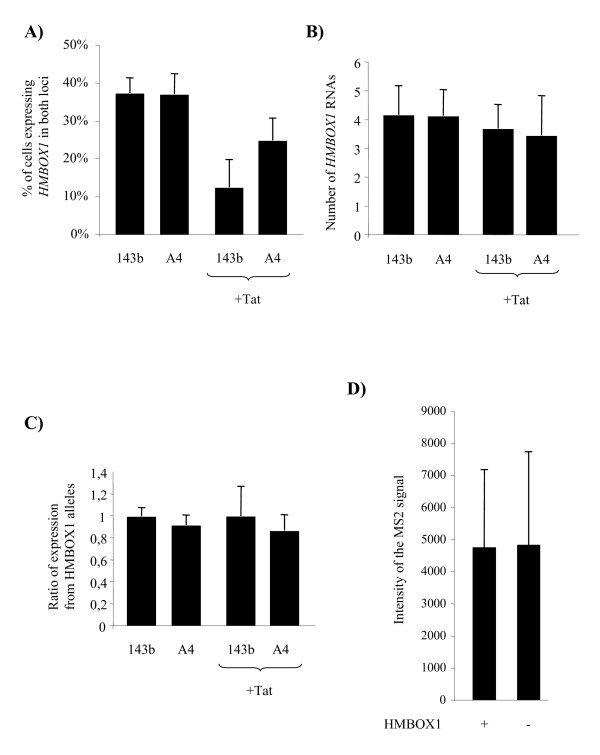
**A) The number of cells expressing *HMBOX1 *in both loci before and after Tat-EGFP transfection is shown. B)** The number of *HMBOX1 *RNAs on the transcription spots before and after Tat-EGFP transfection is shown. **C)** The ratio between the intensity of the hybridization signals on the transcription spots of the *HMBOX1 *alleles is shown. **D) **The intensity of the MS2 hybridization signal on HIV-Intro transcription spots is shown both on a *HMBOX1 *active and inactive background.

*HMBOX1 *expression was low and could not be detected by a single probe. Therefore a mixture of eight oligonucleotides, distributed in the first and fourth exon and in the second intron before and after the integration (Figure 1), were designed to detect the nascent unprocessed RNA transcripts of *HMBOX1 *(Table 1). As shown in figure 5A, in parental HOS-143b two spots of equal intensity were clearly visible in 37.2% of nuclei indicating that the *HMBOX1 *gene is expressed from both alleles, but only in a fraction of the asynchronous population of cells. This is not surprising since there is ample variation of the number of alleles/nucleus detected by this method, depending on how robust is gene expression and how efficient is the processing of the RNA; both contribute to the level of RNA at the site of transcription at steady state [35]. It is however unlikely that detection was lowered by scarce accessibility of the probe to the RNA since positive cells showed invariably two alleles of equal intensity, where in the case of technical problems there would be a higher proportion of single-allele expressing cells. The same was observed in derivative HOS_A4 cells where 36.8% of nuclei showed biallelic expression of the *HMBOX1 *gene (Figure 5C). The number of nascent RNAs present on the transcription site at steady-state was calculated to be significantly similar in both cell lines: 4.13 ± 1.02 for HOS_143b and 4.11 ± 0.91 for HOS_A4 (p value = 0.18) (Figure 6B). Most importantly, the ratio between the intensity of the signal of the two loci was invariably close to 1 in both cell lines demonstrating bi-allelic expression of the *HMBOX1 *gene with comparable levels (Figure 6C).

**Table 1:** Primers for RT-PCR and probes for RNA FISH used in this work.

Name	Sequence 5' > 3'
**PRIMERS FOR RT-PCR**	
HIV_UP5	GGCGAGCCCTCAGATCCTGC
HIV_START	GGGTCTCTCTGGTTAGACCAGATCTGAGCC
HIV_RT	AGTCGCCGCCCCTCGCCTCCTGC
HIV_SPLICED	GGATTAACTGCGAATCGTTCTAGC
HIV_NUC	CGTCTGTTGTGTGACTCTGGTAACT
HMBOX_E1s	GTCTCTTTCCTCACTTCTTTCT
HMBOX_E1a	TCACAGTTTCCAGAACTCCAC
HMBOX_E2s	GGAATGGAACAGTGAAGAAGCA
HMBOX_E3s	AATGGTAGATAACGCAGATCATC
HMBOX_E3a	ACCACTGGAAAGGAACTAAGCA

**PROBES FOR RNA FISH**	
HIV_MS2	AxGTCGACCTGCAGACAxGGGTGATCCTCAxGTTTTCTAGGCAATxA
HMBOX_E1a	AxAGTTTCCAGAAxTCCACACCGGAGACCCCACxTCCAGGATTCAAACCxT
HMBOX_E1b	CxCAGCGTCCGCxCACTTCCTCCCCAAAACCCCxCCAAAAAAATTGTTxT
HMBOX_I2a	AxGGTTGGTATAAACACAxAAAGCATGGTGGTxGTCTGGAGCTGGGGTTxA
HMBOX_I2b	AxTGCAGTGAGCCATGAxCACACCACAGTACxACAGCCTGGGTGATGAAxA
HMBOX_I2c	AxATTGCTGTCCTAAxCAGACTGCACCTGTGGxGTGGCTCTGACTGGTxA
HMBOX_I2d	AxGGTATGGTGGCAAAxCGACTCCCCCAGxACAACCACCAGAATATCAGxA
HMBOX_E4a	AxACGCCGAAGTCGCxGAAGCAGATCTATCTGCxCTATGGTAAATCTGGxA
HMBOX_E4b	AxATAACTGTTGCTAGGxGACGGGGACATTCCCGAAxGCTGCGTCTGTxA

Modified aminoallyl thymidines in the probes are indicated with "x". The sequence of one binding site for MS2 is underlined in the HIV_MS2 probe.

**Figure 7:**
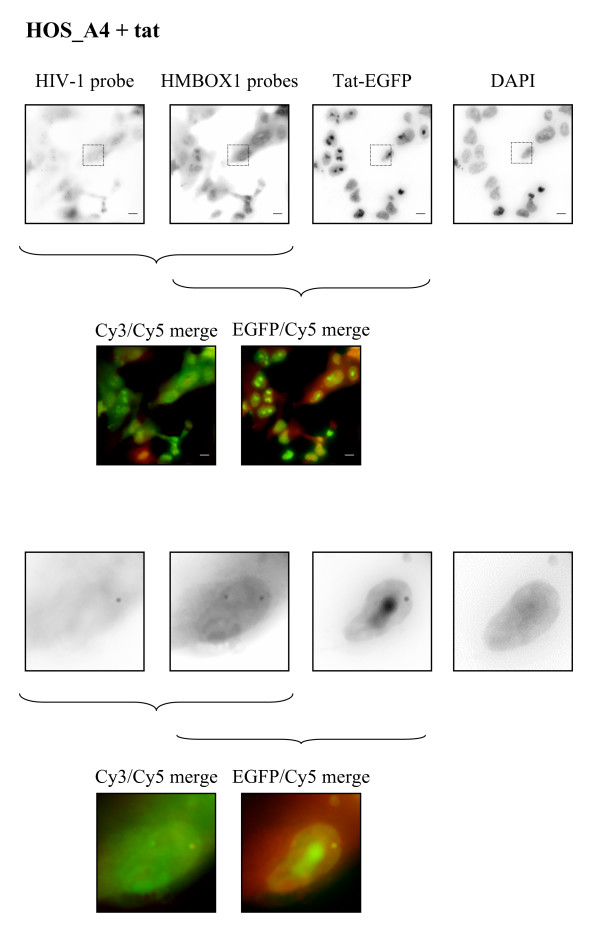
**FISH for *HMBOX1 *and HIV-Intro RNA on HOS_A4 cells after transfection of Tat-EGFP**. Merged images are shown in color both for the large fields and for the zoomed figures (bar = 10 μm).

Next we investigated the effect of Tat transfection. Consistently with what has been observed with RT PCR analysis, the number of Tat-transfected nuclei showing expression of the two *HMBOX1 *loci decreased (Figure 6A). This difference was significant in both cell lines (p value = 1.34 × 10^-23 ^for HOS_143b and p value = 2.63 × 10^-4 ^for HOS_A4). Interestingly, in those cells where both spots were detected, the number of RNAs and the ratio of the *HMBOX1 *alleles were not affected (Figure 6B and Figure 6C). HIV transcripts instead were present in most (93%) of Tat-EGFP transfected cells, consistent with transactivation of the viral LTR in the clonal population (Figure 7). Interestingly, most cells that express the vector do not show expression of *HMBOX1 *in both alleles, indicating that the effect on the expression of *HMBOX1 *was dependent on the expression of Tat and not on the transactivation of the provirus. Even more strikingly, in 11% of Tat-EGFP transfected nuclei both *HMBOX1 *alleles and the proviral transcript were active (Figure 7). In this subset of cells the ratio between *HMBOX1 *alleles was also close to 1 and the intensity of the proviral signal comparable to that of cells where there was no *HMBOX1 *expression. In fact, as shown in figure 6D, transcription of HIV-1 was not significantly affected by allele-specific *HMBOX1 *transcription (p value = 0.91). Hence, there are conditions where intragenic transcription of HIV-1 can occur in the presence of transcription of the host gene.

## Discussion

Integration of HIV-1 in host chromatin is a crucial event for viral pathogenesis. Chromatin control of provirus gene expression has been postulated to be a major determinant of post-integration latency that is the cause of failure to eradicate HIV-1 infection by current antiretroviral regimens [1-3]. In addition, development of lentiviral vectors for gene therapy requires that endogenous genes shouldn't be affected by the integration event. However, recent evidence suggests that down-modulation of HIV-1 expression occurs also within active genes, in the absence of a repressive chromatin context. A cell line harboring a Tat-inducible HIV-1 vector integrated within the *HMBOX1 *endogenous gene has been engineered in this work. This allowed the detailed investigation of the reciprocal influence of HIV-1 and *HMBOX1 *expression both with or without Tat induction.

As a result of the double selection procedure, HOS_A4 showed a basal level of HIV-1 RNA by RT PCR that could be assigned to *HMBOX1 *read-through transcription across the silent HIV-1 provirus since neither RNA was evident in FISH (Figure 5C) nor ECFPskl could be detected in the cytoplasm (Figure 3A). Tat overexpression, while increasing HIV-1 expression as expected, also reduced the level of expression of *HMBOX1 *in both alleles. Besides its essential role in *trans-*activating HIV-1 transcription, Tat is known to regulate key host cell functions, primarily at the level of transcription. For example, Tat down-regulates MHC class II by preventing the interaction of cyclin T1 with the class II transactivator CIITA [36]. It is conceivable that Tat, being able to interact with a variety of host factors required for HIV transactivation [13,37], at the same time pulls these factors away from specific host genes, altering transcription from these promoters. Genome-wide expression profiling indeed revealed that Tat overexpression resulted in down-modulation of many cellular genes, possibly through targeting of general factors such as the SWI/SNF chromatin remodeling complex and the p300 acetyltransferase [38,39]. Hence, *HMBOX1 *adds to the list of genes being down modulated by Tat overexpression. It is possible that during the establishment of the HOS_A4 cell line there has been a positive selection of integration loci where Tat induced repression of transcription. If expression of the endogenous gene interferes in *cis *with the expression of the provirus, the net result of Tat induction would have been of increased LTR-driven expression due also in part to the decrease of *HMBOX1 *expression. However, the number of HIV-1 nascent RNAs in Tat-transfected HOS_A4 cells that were negative for *HMBOX1 *expression was similar to the number of those where instead *HMBOX1 *was active (Figure 6D).

Another important finding of this work was that in some cells expression of the endogenous *HMBOX1 *gene and of the provirus coexisted at the same transcription site. This finding could not be anticipated since it was believed that transcriptional interference should have occurred. Transcriptional interference is the suppressive influence of one transcriptional process directly and in *cis*, on a second transcriptional process [40,41]. Several combinations of the disposition of the two transcription units produce different effects [42]. For example, two promoters firing in opposite orientation would end up in collision of the two converging transcription elongation complexes. In HOS_A4 cells instead, the two promoters elongate in the same direction allowing a possible transcriptional interference through occlusion of the downstream promoter. In such model transcription from the upstream *HMBOX1 *promoter should transiently preclude the occupation by RNAPII and/or associated transcription factors of the downstream LTR promoter. Alternatively, the LTR could pose a roadblock to the progress of the transcription-elongation complex firing from the *HMBOX1 *promoter resulting in its inhibition of expression from one allele.

The observation that in the absence of Tat transcription both *HMBOX1 *alleles were equally expressed while expression from the viral LTR remained undetectable might indicate that transcription elongation of RNAPII across the *HMBOX1 *gene occluded the viral LTR. The situation changed dramatically in the presence of Tat. Transcription from the viral LTR is switched on as shown in RT PCR as well as demonstrated by the appearance of the viral transcript in RNA FISH. As discussed previously, Tat repressed *HMBOX1 *expression while allowing provirus transcription. However, in a subset of cells, expression of both genes coexisted. Recent data also analyzed HIV proviral gene expression from within a cellular gene [43]. Work by Peterlin's group showed transcriptional interference occurring from elongating polymerase firing from the host gene [44], whereas work from Silicano's laboratory showed that elongating polymerase from the host gene could enhance HIV transcription when orientated in the same direction [45]. It is difficult to compare directly these data with those presented here since different cell lines and host cell genes were studied. However the findings presented in this work show an alternative situation that will require further analysis to understand the molecular basis of the phenomenon.

## Conclusion

In this work HIV-1 gene expression was studied from within the endogenous gene *HMBOX1*. Transcriptional Tat-transactivation of the viral LTR resulted in up-regulation of HIV-1 transcription while it repressed HMBOX1 gene expression in both alleles, independent of vector integration. Hence, it could be proposed that HIV-1 genome insertion in genes repressed by Tat could be an advantage for the virus allowing its transactivation from a low-transcribing endogenous gene. It has also been observed that both HIV-1 and *HMBOX1 *gene expression may occur at the same genomic location in the same cell, allowing speculation on the lack mutual interference between transcription units.

## Methods

### Plasmids and cells

Plasmid pHIV-Intro was derived from the plasmid pEV731 [27] by cloning 24 MS2 repeats into the NotI restriction site. A cassette encoding for ECFP with the peroxisome localization signal Ser-Lys-Leu (skl) was inserted between the ClaI and XhoI sites, the IRES from EMCV and the thymidine kinase from HSV-1 were cloned in the XhoI site [46]. To obtain cells transduced by the HIV-Intro lentiviral vector expressing the HSV-TK gene we exploited a protocol for negative and positive selection of TK- cells. HEK-293T cells were transfected with the vector plasmid pHIV-Intro together with the packaging plasmids as previously described [26]. The supernatant was filtered and used to transduce human bone osteosarcoma TK- cells (HOS 143b, ECACC n. 91112502). Next day cells were treated with ganciclovir at 50 μg/ml. Surviving cells were expanded and then treated with 2.5 μg/ml of GST-Tat to induce LTR expression as described previously [34,47]. The following day cells were incubated in hypoxanthine, aminopterin and thymidine (HAT) medium and then cloned by colony picking and expansion in complete DMEM medium. Colonies were visually scored for low basal level of ECFP expression and to be highly inducible by GST-Tat by fluorescence microscopy. The number of integrations was assessed by southern blotting with a probe for ECFP and the cloning of the integration sites was obtained by inverse PCR essentially as described [26]. Briefly, genomic DNA was digested with BamHI (cleavage site within HIV-Exo-MS2 × 24), and the resulting products were circularized with DNA ligase. The product of two nested PCRs performed with primers pointing outwards from the vector was cloned and sequenced.

Plasmids expressing Tat and MS2 have been described previously [30,48]. Tat and Rev were fused to pDsRedMonomer-N1 (Clontech) by PCR.

### Allele-specific RT-PCR

Total RNA was extracted as described by the kit (Qiagen, RNA easy). Reverse transcription was performed with M-MLV RT (Invitrogen) using random primers. Amplification of the cDNA was conducted in the presence of CyberGreen™ (Applied Biosystems) and monitored on AbiPrism 7000 (Applied Biosystems). Specific primers are shown in Table 1.

### Quantitative RNA fluorescent in situ hybridization (RNA FISH)

In situ hybridization was performed essentially as previously described [30]. Cells were fixed with 4% PFA pH7.4 and permeabilized overnight in 70% ethanol. Formamide concentration was 40% for *HMBOX1 *probes and 20% for MS2. The amino-allyl thymidine modified oligonucleotide probes (Table 1) have been synthesized by J-M Escudier (Platforme de synthèse d'Oligonucléotides modifiés de l'Interface Chimie Biologie de l'ITAV, Toulouse, France). Probes were labeled with Cy3 or Cy5 (Cyn monoreactive dye, Amersham). For quantitative measurements stacks of 21 planes were acquired at bin = 2 with steps of 0.5 μm in the z-axis using a wide-field Leica DMRI inverted microscope (63× objective, NA 1.3) controlled by Metamorph (Universal Imaging). Digital images were collected using a CoolSnap K CCD camera (Roper scientific). The three-dimensional deconvolution and reconstruction was performed with the ImageJ plug-in "Iterative Deconvolve 3D". The total light intensity at the transcription site was calculated and divided for the number of planes and the number of molecule were computed from a calibration curve of the probes in solution [30].

## Competing interests

The authors declare that they have no competing interests.

## Authors' contributions

ADM and CB carried out the RT-PCR, ADM and PM carried out the *in situ *hybridization and quantitative analysis, AK, CV and AM prepared and characterized the cell line, AM contributed to the experimental design and coordination of the study, data analysis, as well as to writing the manuscript. All authors have read and approved the final manuscript.
